# A systematic review of COVID-19 and the presentation of avoidant/restrictive food intake disorder and avoidant/restrictive food intake disorder-like symptoms

**DOI:** 10.1192/bjo.2023.655

**Published:** 2024-03-04

**Authors:** Kristen Maunder, Oscar Markey, Rachel Batchelor, Fiona McNicholas

**Affiliations:** Department of Child and Adolescent Psychiatry, University College Dublin, Ireland; Department of Liaison Child and Adolescent Psychiatry, Tallaght University Hospital, Ireland; and Department of Child and Adolescent Psychiatry, Linn Dara Child and Adolescent Mental Health Services, Dublin, Ireland; Department of Psychology, Universiteit Leiden, The Netherlands; Department of Psychology, Trinity College Dublin, Ireland; and Department of Child and Adolescent Psychiatry, Lucena St John of God's Child and Adolescent Mental Health Services, Dublin, Ireland; Department of Psychology, Oxford Institute of Clinical Psychology Training and Research, UK; and Department of Psychology, Oxford Health NHS Foundation Trust, UK; Department of Child and Adolescent Psychiatry, University College Dublin, Ireland; Department of Child and Adolescent Psychiatry, Lucena St John of God's Child and Adolescent Mental Health Services, Dublin, Ireland; and Department of Liaison Child and Adolescent Psychiatry, Children's Health Ireland at Crumlin, Ireland

**Keywords:** Avoidant/restrictive food intake disorder, eating disorders, COVID-19, prevalence, clinical presentation

## Abstract

**Background:**

The adverse effects of COVID-19 and the associated restrictions on eating disorder populations have been discussed in recent literature. However, little is known about the presentation of cases with avoidant/restrictive food intake disorder (ARFID) during this period.

**Aims:**

To explore the extent of the literature on the presentation of ARFID, and ARFID-like cases, during the COVID-19 pandemic.

**Method:**

Cochrane Library, CINAHL (EBSCO), PsycINFO (EBSCO), EMBASE (Ovid) and Medline (Ovid) were searched for publications between March 2020 and May 2023. Google Scholar and reference lists were hand searched. At least two reviewers independently screened each paper. Narrative synthesis was used.

**Results:**

Seven papers were included: four case reports and three cohort studies (total ARFID sample of 46). Included papers were assessed as having high (*n* = 3) or moderate (*n* = 4) quality. Findings did not suggest an increase in ARFID cases during the COVID-19 pandemic, although it is unclear if this is because of a lack of impact or underrecognition of ARFID. A need for a multidisciplinary approach to differentiate between ARFID and organic causes of ARFID-like presentations (e.g. gastrointestinal effects of COVID-19) was highlighted.

**Conclusions:**

Publications specifically pertaining to ARFID presentations during the COVID-19 pandemic have been few. Papers found have been of small sample sizes and lack subanalyses for ARFID within broader eating disorder samples. Continued surveillance is needed to evaluate any COVID-19-specific effects on the development, identification, treatment and outcomes of ARFID.

COVID-19, an infectious disease caused by SARS-CoV-2 virus, reached pandemic status in March 2020. The COVID-19 pandemic led to several restrictions, including social distancing measures, quarantining and limitations on social interactions and access to care, resulting in concerns surrounding mental health. Since the beginning of the pandemic, increased levels of psychological distress, anxiety and depression have all been reported across age groups.^1–3^

Eating disorder research has identified several risk factors for disordered eating in the context of COVID-19. For some, disrupted routines including eating- and exercise-related habits, reduced peer-related activity and more time spent on social media, with the possibility of weight-stigmatising messages, have contributed to unhealthy and individual focus on eating and exercise.^4–6^ Moreover, non-specific vicarious trauma and stress, and COVID-19 anxiety, may have increased general vulnerability alongside decreased protective factors such as socialisation and healthy coping mechanisms.^4,5^ COVID-19 has exacerbated barriers to eating disorder assessment and treatment, with longer waiting lists for assessments and treatment, limited face-to-face care and changes in the care that families and carers receive from eating disorder services to support their loved ones.^7–9^ Such restrictions have also contributed to typically delayed presentation for assessment, with the subsequential risk of increased severity at the eventual point of diagnosis and treatment.^10^

The COVID-19 pandemic has seen an increase in eating disorder helpline contacts, out-patient care-related inquiries and hospital admissions for eating disorders following lockdowns.^8,11,12^ Increased eating disorder symptoms such as restrictive eating, hyper-exercising, binge-eating and purging have also been reported within the general public as well as those with eating disorders,^13–15^ and across a range of groups including children, adolescents, university students and adults worldwide.^13,16–19^ Atypical presentations in children have also been reported.^20^

Diagnostic incidence rates of eating disorders have also increased during the COVID-19 pandemic, with a study utilising 5.2 million health records reporting diagnostic incidence to be 15.3% higher in 2020 overall compared with the previous 3 years, with the rate increasing steadily throughout 2020 following the first few months.^21^ A recent systematic review that included 53 studies and 36 465 individuals with eating disorders demonstrated increased symptoms for anorexia nervosa, binge eating disorder and bulimia nervosa.^22^

## Avoidant-restrictive food intake disorder and the COVID-19 pandemic

Compared with other eating disorders, little is known about how the circumstances of the COVID-19 pandemic have affected the prevalence and presentation of avoidant/restrictive food intake disorder (ARFID). As described in the DSM-5, ARFID is characterised by avoiding certain foods or types of food and/or having restricted intake in terms of overall amount eaten.^23^ Such avoidance and/or restriction of food intake can be for several reasons, most commonly sensory-based avoidance or restriction, concerns about aversive consequences of eating and low interest in eating; individuals may experience one or more of these reasons.^23,24^

To meet diagnostic criteria for ARFID under the DSM-5, individuals must have one or more of the following: significant weight loss (or failure to achieve expected weight gain or faltering growth in children), significant nutritional deficiency, dependence on enteral feeding or oral nutritional supplements, and marked interference with psychosocial functioning.^23^ Such difficulties cannot be better explained by lack of available food or cultural sanctions, and are not attributable to a concurrent medical condition or better explained by another mental health condition.^23^

ARFID does not occur alongside cognitions typically associated with anorexia nervosa and other eating disorders; for example, there is no fear of becoming fat, and no dysmorphia.^23,25^ In addition, individuals with ARFID do not purge, intensely exercise or in any other way persistently attempt to lose weight.^23,25^ ARFID is most prevalent in early childhood, although can occur across the lifespan, with cases commonly being comorbid with autism and reported most often in males.^26^ Restrictive eating behaviours are common in childhood, and it is important to be able to distinguish transient difficulties or changed eating patterns from ARFID, given the recognition that early identification and treatment of eating disorders, including ARFID, are associated with better outcomes.

Estimated prevalence of ARFID has been reported as between 5 and 14%.^27^ However, a recent systematic review, despite endorsing important contributions that epidemiological studies have made to our understanding of the disorder, concluded that prevalence estimates remain unknown given methodological limitations of studies conducted.^28^

The aim of this review is to examine what has been written in the literature with regards to presentation of cases with ARFID during the COVID-19 pandemic.

## Method

### Protocol

This systematic review was conducted in line with the Preferred Reporting Items for Systematic Reviews and Meta-Analysis (PRISMA) 2020 statement.^29^ A search was conducted on the international database of Prospective Register of Systematic Reviews (PROSPERO) in January 2022 to confirm that a similar review had not previously been registered. The original protocol of this systematic review was registered on PROSPERO in March 2022 (identifier CRD42022308143).

### Search strategy

A systematic search of published studies on ARFID and COVID-19 was performed from 11 March 2020, which was when the World Health Organization (WHO) declared COVID-19 a pandemic, until the search date (4 May 2023). The following databases were searched: Cochrane Library, CINAHL (EBSCO), PsycINFO (EBSCO), EMBASE (Ovid) and Medline (Ovid).

The search algorithm included the following terms: ‘avoidant/restrictive food intake disorder’ OR ‘avoidant eating’ OR ‘restrictive eating’ OR ‘food refusal’ OR ‘calorie restrict*’ AND COVID-19 OR COVID* OR corona*. The search terms were entered into the title, abstract and keyword fields on Cochrane Library, CINAHL (EBSCO), PsycINFO (EBSCO), EMBASE (Ovid) and Medline (Ovid) (see Supplementary Materials available at https://doi.org/10.1192/bjo.2023.655 individual database search terms). Keywords and terms from the search strategy were also entered in Google Scholar to search for additional papers, and reference lists of included studies were manually searched.

### Inclusion and exclusion criteria

Study inclusion criteria were as follows: published in a peer-reviewed journal; included one or more individual(s) of any age with an ARFID diagnosis as described in the DSM-5 or ICD-11, likely to meet ARFID criteria if they were to be assessed, found to meet ARFID criteria retrospectively or with any ARFID-like presentations; data collection occurred during the COVID-19 pandemic; and used qualitative, quantitative or mixed methods, including single case studies and case series presenting quantitative data regarding the prevalence, presentation or course of ARFID.

Study exclusion criteria were as follows: only described avoidant or restrictive eating in the context of a different eating disorder; focused only on ARFID treatment; not available in the English language; unpublished research; had not undergone a peer review process and no reference to COVID-19.

### Screening and data extraction

All references identified through the searches were imported into a Microsoft Excel for Windows database. The initial screening of the titles and abstracts of all items was undertaken by at least two reviewers independently. If an article was deemed relevant, the full-text manuscript was obtained and further screened by two authors (O.M. and R.B.). Any disagreement regarding eligibility for inclusion was resolved through further discussion between all authors from the research team.

A structured data extraction sheet on Microsoft Excel was developed and pilot tested to extract relevant data from the included reviews. Reviewers O.M. and R.B. independently extracted data by using the form; another reviewer (K.M. or F.M.) verified the data extracted such that all extracted data had been independently verified. Data extraction began on 1 May 2022 and was updated on 15 June 2023.

### Quality assessment strategy

Two reviewers (O.M. and R.B.) independently assessed and cross-checked the quality of each paper, using Joanna Briggs Institute (JBI) critical appraisal tools.^30^ Each appraisal has a set of (responses: yes, no, unclear, not applicable). ‘Yes’ indicates a low risk of bias and ‘no’ or ‘unclear’ denotes a high risk of bias, and the results are expressed as the frequency of each classification of the evaluation parameters. The quality of each study was categorised as high, moderate or low.

### Data synthesis

The data was not sufficiently homogenous for quantitative pooling (i.e. a meta-analysis was not appropriate because of the heterogeneity of study designs and outcome assessment methods). Therefore, a narrative synthesis approach was conducted from the full-text articles. For synthesis, studies were grouped by study type (i.e. case reports and cohort studies). Other participant characteristics, such as age and comorbid conditions, were also considered and presented (Table 1).

**Table 1:** Summary of study characteristics and findings

Author (year)	Study type and country	Age (years) and gender	Full cohort sample and timeframe	ARFID sample size	ARFID diagnosis criteria provided	Summary and any putative link with COVID-19
Borriello et al (2021)^32^	Case report, in-patient psychiatry setting, Italy	20, female	2-week onset, 4-week hospital admission	*N* = 1	NDCP, ARFID diagnosis by inference from clinical symptoms (mentioned the DSM-5 for borderline personality disorder, but not specifically for ARFID)	COVID-19 induced gastrointestinal symptoms, responded only when became COVID-19 negative. Highlights difficulties differentiating between psychiatric illness and COVID-19 gastrointestinal effects
Cao et al (2022)^34^	Case report, medical admission, USA	3, male	1-month food refusal before admission. Days in treatment not specified	*N* = 1	NDCP, ARFID diagnosis by inference from clinical symptoms (the DSM-5 cited, but not stated that it was used to diagnose)	COVID-19 disruption of routine, causing escalating stress levels and acute food refusal
Sakamoto et al (2021)^36^	Case report, medical admission to paediatric medical centre and out-patient psychiatry clinic, Japan	8, male	2–3 months (unclear)	*N* = 1	NDCP, ARFID diagnosis by inference from clinical symptoms	Risk of COVID-19-phobia-related reduced food intake/weight loss among youth with autism
Yazdani et al (2022)^37^	Case report, admission to paediatric medical centre, USA	18, female	Increased exercise over 2-month period, resulting in weight loss and restrictive diet from gastrointestinal symptoms	*N* = 1	ARFID diagnosis provided along with evidence of clinical symptoms	Attributed increased exercise to the inability to participate in sports because of COVID-19. Resulted in weight loss, gastrointestinal symptoms and subsequent restrictive eating in the context of ARFID
Otto et al (2021)^38^	RCNCS, restrictive eating disorder related medical admissions, USA	10–23	Pre-COVID-19: March 2017 to March 2020, *n* = 146, 14 males, 129 females, 2 transgender males, 1 transgender female. During COVID-19: April 2020 to March 2021, *n* = 102, 8 males, 92 females, 2 non-binary	Pre-COVID-19: *n* = 13 (8.9%). Post-COVID-19 onset: *n* = 7 (6.9%)	No	Increased numbers of patients with ARFID presenting post-COVID-19 onset than before (although reduced proportion of patients presenting with ARFID compared with overall restrictive eating disorders during COVID-19)
Spettigue et al (2021)^39^	RCNCS, specialist eating disorder service, Canada	10–18	Pre-COVID-19: 1 April to 31 October 2019, *n* = 48. During COVID-19: 1 April to 31 October 2020, *n* = 43	Pre-COVID-19: *n* = 7 (16.3%). Post-COVID-19 onset: *n* = 7 (14.6%)	No (mentioned the DSM-5 for anorexia nervosa, but not specifically for ARFID)	No change in proportion of ARFID post-COVID-19 onset, and the majority of ARFID cases did not perceive COVID-19 to be triggering
Takakura et al (2022)^40^	RCNCS, specialist eating disorder service, Japan	11–68	Pre-COVID-19 onset: 1 April 2019 to 6 April 2020, *n* = 86. Post-COVID-19 onset: 7 April 2020 to 31 March 2021, *n* = 62	Pre-COVID-19 onset: *n* = 3 (3.5%). Post-COVID-19 onset: *n* = 5 (8.1%)	DSM-5	Increase in proportion of ARFID post-COVID-19 onset

ARFID, avoidant/restrictive food intake disorder; NDCP, no diagnostic criteria provided; RCNCS, retrospective case note cohort study.

## Results

**Fig. 1:**
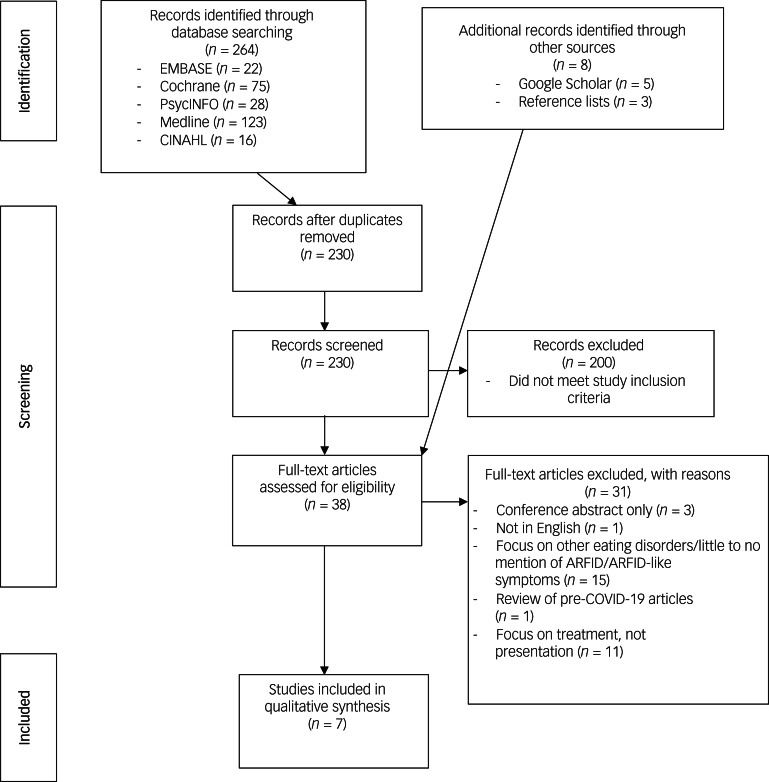
Preferred Reporting Items for Systematic Reviews and Meta-Analysis (PRISMA) flow chart of the systematic literature review. ARFID, avoidant/restrictive food intake disorder.

The search strategy yielded 264 citations. Citations were screened for duplicates, and 34 were removed. The title or abstract of the remaining 230 articles were screened and 200 were excluded. It was evident from the title or abstracts that these articles were study protocols, were not a peer-reviewed published paper, were not available in the English language, only included data that was collected before COVID-19, had a study population specific a different eating disorder (e.g. all participants have a diagnosis of anorexia nervosa), examined prescribed dietary treatments or were not relevant to the topic being studied. Of the remaining 30 articles, the full texts were read and assessed for eligibility; 25 were excluded and five were included. The reasons for exclusion after full-text reading are listed in Fig. 1. Google Scholar and reference lists of included studies were hand searched, with an additional eight papers identified and screened; six were excluded and two were included. A PRISMA flow diagram of this process is provided in Fig. 1. The final selection included four case reports and three cohort studies. Table 1 provides details on the seven articles included in this systematic review.

### Quality appraisal

The JBI critical appraisal checklist was used to assess the methodological quality of each study.^31^ The overall level of bias as assessed by the JBI of each of the included studies are presented in Tables 2 and 3. Based on the JBI checklist, three studies (42.9%) were considered of high quality, whereases four studies (57.1%) was considered of moderate quality (see Tables 2 and 3). Overall, it was felt that most studies were representative of the target population, and that study participants were recruited in an appropriate way and described in detail. Generally, there was an absence of reporting of confounding factors. Given that this research area is still in an early stage, no papers were excluded based on their quality rating, although all papers were further considered in the context of limitations.

**Table 2:** Joanna Briggs Institute checklist for case reports

	Case reports			
	Sakamoto et al (2021)^36^	Borriello et al (2021)^32^	Cao et al (2022)^34^	Yazdani et al (2022)^37^
Were patient's demographic characteristics clearly described?	Yes	Yes	No	Yes
Was the patient's history clearly described and presented as a timeline?	Yes	No	Yes	Yes
Was the current clinical condition of the patient on presentation clearly described?	Yes	Yes	Yes	Yes
Were diagnostic tests or assessment methods and the results clearly described?	No	Yes	No	Yes
Was the intervention(s) or treatment procedure(s) clearly described?	No	Yes	Yes	Yes
Was the post-intervention clinical condition clearly described?	Yes	Yes	No	No
Were adverse events (harms) or unanticipated events identified and described?	No	Yes	Yes	No
Does the case report provide takeaway lessons?	Yes	Yes	Yes	Yes
Overall appraisal (<5 = low, 5–7 = moderate, ≥8 = high)	5 = moderate	7 = moderate	5 = moderate	6 = moderate

**Table 3:** Joanna Briggs Institute checklist for cohort studies

	Cohort studies		
	Otto et al (2021)^38^	Spettigue et al (2021)^39^	Takakura et al (2022)^40^
Were the two groups similar and recruited from the same population?	Yes	Yes	Yes
Were the exposures measured similarly to assign people to both exposed and unexposed groups?	Yes	Yes	Yes
Was the exposure measured in a valid and reliable way?	Yes	Yes	Yes
Were confounding factors identified?	No	No	No
Were strategies to deal with confounding factors stated?	No	No	No
Were the groups/participants free of the outcome at the start of the study (or at the moment of exposure)?	Yes	Yes	Yes
Were the outcomes measured in a valid and reliable way?	Yes	Yes	Yes
Was the follow-up time reported and sufficient to be long enough for outcomes to occur?	Yes	Yes	Yes
Was follow-up complete, and if not, were the reasons to loss to follow-up described and explored?	Yes	Yes	Yes
Were strategies to address incomplete follow-up utilised?	Not applicable	Not applicable	Not applicable
Was appropriate statistical analysis used?	Yes	Yes	Yes
Overall appraisal (<5 = low, 5–7 = moderate, ≥8 = high)	8 = high	8 = high	8 = high

### Case reports

Borriello and colleagues^32^ presented the case of a 20-year-old Italian woman admitted to a psychiatric ward (Milan) following presentation to the emergency department with fever (38°C), severe weight loss (7 kg in 2 weeks, resulting in a drop of her body mass index (BMI) from 24.2 to 21.8 kg/m^2^) and recurring depression. She had a history of ARFID (present since childhood) and borderline personality disorder according to the DSM-5, as well as self-harm and depression. She also had prior disordered eating behaviour characterised by restrictive eating and self-induced vomiting, and on admission, endorsed a high drive for thinness on the Eating Disorder Inventory-2.^33^ She tested positive for COVID-19 and 2 days into her admission she developed significant associated gastrointestinal symptoms of nausea, vomiting, loss of appetite and diarrhoea. These were viewed to be secondary to her COVID-19 infection. The authors noted that her depressive symptoms improved rapidly; however, her overall gastrointestinal symptoms, nausea, poor food intake and liver abnormalities persisted for 35 days and, despite treatment, only resolved when she became COVID-19 negative. The authors highlighted the diagnostic complexity in this case, with a prior diagnosis of psychiatric illness and new emergent symptoms in the presence of a medical condition, COVID-19 (i.e. difficulties existed in differentiating between symptoms attributed to psychiatric conditions and those attributed to COVID-19 complications – in this case, a presentation of ARFID-like gastrointestinal symptoms owing to COVID-19). This shows the complexity of diagnosis and treatment, and emphasises the importance of the correct use of evaluation scales complementary to a diagnosis, as well as taking a multidisciplinary approach.

Cao and colleagues^34^ presented the case of a 3-year-old male child with autism and a longstanding history of food selectivity based on taste/texture. He presented to the emergency department in the USA, following acute food refusal of 1 month's duration and difficulty walking (‘wobbling’), diarrhoea and decreased activity levels. At the time of presentation, his food intake was limited to six to eight cups of diluted drink and occasional snacks, such as small bag of chips or cake. Prior food selectivity included a preference for cakes, muffins, crackers, peanut butter, chips, and nuggets. Parents attributed his escalating stress levels to be linked with COVID-19 restrictions and cancellation of usual activities (routine disruption). His weight reduced from 13.8 to 12.4 kg and his BMI decreased from the 10th to <1st percentile. It is unclear if ARFID was officially diagnosed; he was considered to meet the American Society for Parenteral and Enteral Nutrition (ASPEN) criteria for severe acute undernutrition,^35^ likely secondary to ARFID (but not further defined). He was also tachycardic to 120 beats per minute and malnourished in appearance, with bilateral angular cheilitis and capillary refill time of 3 s. Extensive laboratory evaluation demonstrated severe electrolyte abnormalities and dehydration, micronutrient deficiencies, hypochloraemia and elevated bicarbonate. He initially had low potassium and hypokalaemia (placing him at high risk of cardiac arrythmias), and high creatine kinase, suggestive of hypokalaemia-induced rhabdomyolysis. He was treated with intravenous fluids (electrolyte therapy) for his dehydration and commenced on nasogastric feeding, gaining 200 g per day, and began tolerating increasing amounts of oral intake. He subsequently developed hyperkalaemia, believed to be secondary to muscle breakdown; after more intravenous fluids and a single dose of furosemide, his potassium levels normalised. He was discharged with micronutrient supplementation, nasogastric feeds and follow-up appointments. The authors highlighted that this is the first documented paediatric case of rhabdomyolysis secondary to hypokalaemia from severe undernutrition. Although the authors do not specifically state a diagnosis of ARFID, they conclude he met criteria for severe acute undernutrition. The authors highlighted the increased risk of eating difficulties (e.g. food selectivity and a preference for the same food) among individuals with autism, particularly in the context of stressors such as the pandemic, and the importance of assessing for rhabdomyolysis in patients with hypokalaemia, as symptoms of hypokalaemia-induced rhabdomyolysis can be subtle and lead to life-threatening complications.

Sakamoto and colleagues^36^ reported the presentation of an 8-year-old Japanese male child with no history of eating disorders, who developed COVID-19 phobia leading to ARFID-like symptoms. The patient became phobic following media representation of the pandemic. and he stopped attending school or socialising, increased his mask wearing and became insistent of others maintaining high safety measures. Subsequent fear of viral contamination from food culminated in his refusal to eat or drink (or swallow his saliva) over a 3-week period, with resultant life-threatening dehydration requiring medical admission and management. A standardised semi-structured parental interview and observations confirmed a diagnosis of autism based on the DSM-5 criteria. The total clinical evaluation demonstrated that he had COVID-19 phobia, resulting in ARFID. After medical stabilisation with fluids and nutrition, support focused on psychoeducation of autism and COVID-19 and engaging the child in ensuring the safety of prepared food and reinstatement of routines. Dysphagia improved significantly as his fear faded, and the child was discharged in 2 weeks (care continued in out-patient psychiatry clinic), and his food intake and his body weight returned to pre-onset levels within 2 months. Although the authors did not provide confirmatory diagnostic criteria for ARFID, they highlighted the risk of phobic-related reduced food intake and weight loss in a child with autism following a stressful event such as COVID-19, prompting the consideration of undiagnosed autism.

Yazdani and colleagues^37^ presented the case of an 18-year-old female admitted to a vascular surgery service in the USA with concerns of superior mesenteric artery syndrome in 2020. The psychiatry department were contacted because of the possibility of an eating disorder as a precursor to the illness. The patient mentioned a restricted diet because of nausea and abdominal pain, as well as increased levels of exercise because of the shutdown of organised sports during the COVID-19 lockdown. She had no past medical or psychiatric history. After a computerised tomography scan, gastrointestinal imaging and, finally, an esophagogastroduodenoscopy, the possibility of duodenal obstruction was ruled out. Despite evidence of restrictive eating, the patient did not report any body image concerns or eating disorder cognitions such as fear of weight gain. A diagnosis of ARFID was subsequently given. Weight loss was treated first with a nasogastric tube with initial output of 1 litre, then with refeeding orally over a 4-day period and electrolyte monitoring. Psychiatric services strongly recommended intensive out-patient programmes post-discharge to manage the patient's ARFID, but the patient and family declined. The authors highlighted the lack of awareness of ARFID in the professional and general population, which may have caused the reluctance of the family to accept this diagnosis. In addition, they emphasised the importance of viewing psychiatric diagnoses holistically in the context of the COVID-19 pandemic.

### Cohort studies

Using a retrospective chart review methodology, Otto and colleagues^38^ examined all medical admissions to a children's hospital in the USA, in youth aged 10–23 years for any type of restrictive eating disorders. Those admitted to the psychiatry unit or those with bulimia nervosa or non-restrictive eating disorders were not considered. An interrupted time-series analysis of sequential counts of admissions per month was performed, using the timeframe of March 2017 to March 2021, which showed that eating disorder-related admissions were stable over time before COVID-19 and initially significantly decreased in April 2020 (*P* < 0.001), before significantly increasing over time during the COVID-19 pandemic (*P* < 0.001). More than double the numbers of admissions were made (125 admissions, 102 different patients) during the first 12 months of COVID-19 (April 2020 to March 2021) compared with prior years (March 2017 to March 2020, mean: 56 admissions, total over 3 years: 146 different patients). There was no difference in rates of referrals by eating disorder diagnostic group or clinical demographics, other than a significant increase in those admitted during COVID-19 having private cover (88% *v.* 80% pre-pandemic). Of the total sample, a total of 13 (8.9%) patients had ARFID in the 3 years pre-COVID-19 (*n* = 13, 8.9%) compared with seven (6.9%) in the year during COVID-19, although no diagnostic criteria or clinical details were presented. In their discussion, the authors referred to reasons why/how COVID-19 might have influenced eating disorder symptoms, suggesting that changes (e.g. school closures and cancellation of organised sports) may disrupt eating and exercise routines, with more time to engage in individual programmes, social relationships/isolation, increased time on social media, poorer self-esteem and increased depression. Following COVID-19 restrictions, the authors suggested some change might be a result of youth staying at home, with access to increased parental supervision, and awareness. It is noteworthy that these points referred to restrictive eating disorder populations as a whole, but not ARFID specifically.

Spettigue and colleagues^39^ conducted a retrospective chart review on all patients attending a Canadian paediatric tertiary care service for youth with eating disorders, to examine the impact on referral rate and clinical profile subsequent to COVID-19. The time period covered April to October, 2020 and 2019. There was a slight increase in overall referrals (48 compared with 43 in the pre-pandemic specific timeframe), but no difference in the number of adolescents assessed as having ARFID (seven in 2019 and seven in 2020). In the overall eating disorder sample (i.e. not ARFID specific), higher rates of medical acuity and need for admission were reported, with significantly more (64.6%) being considered medically unstable at admission compared with 34.9% pre-pandemic (*P* = 0.005). Adolescents who first presented for assessment during the pandemic trended toward having a lower percentage of treatment goal weight and higher rates of self-reported impairment, although not significantly. In the overall 2020 sample, 40% felt the pandemic acted as a trigger for their eating disorder. The reported effects of the pandemic included youth being stressed by having to stop playing competitive sports as a result of the lockdown, the stress of worrying about their marks after lessons went online and being bored and lonely at home with nothing to do. Of the youth who perceived COVID-19 to have triggered their eating disorder, duration of symptoms was significantly shorter from onset to assessment (mean: 5.6 months compared) than those who did not (mean: 11.6 months; *P* = 0.007), and although more (78.9% compared with 55.2%) were medically unstable (heart rate below 45 beats per minute; blood pressure <90/50 mmHg; weighed <75% less than ideal body weight; abnormal blood electrolytes), this did not reach statistical significance (*P* = 0.09). In the ARFID sample (*n* = 14), of whom seven presented after pandemic onset, the majority (five out of seven) did not perceive COVID-19 to be a trigger. No demographic information was reported for the ARFID group separately to the overall eating disorder sample, and no specific diagnostic criteria for ARFID were offered.

Takakura and colleagues^40^ extracted data from the medical notes of all females (*N* = 186, although only 148 were included because 38 had missing data) presenting with an eating disorder (diagnosed with the DSM-5) to a psychosomatic medicine department in Japan between April 2019 and March 2021. Clinical characteristics were examined for before (*n* = 86) and after (*n* = 62) onset of the COVID-19 pandemic. The age range of the sample was 11–68 years pre-pandemic and 10–64 years post-pandemic onset; those presenting after pandemic onset were significantly younger overall (median age 22.5 *v.* 19.0 years; *P* = 0.0187). The post-pandemic onset group also had a shorter duration of illness (1.4 *v.* 3.5 years; *P* = 0.005). Age of eating disorder onset or BMI did not differ between the pre- and post-onset groups. In the post-pandemic onset group, a higher rate of ARFID (8.1% *v.* 3.5%) was present, although the number of cases was smaller (three pre-onset, five post-onset). The study also reported that presentations post-onset of COVID-19 had higher levels of maternal care (27.0 pre-onset *v.* 31.5 post-onset; *P* < 0.0001), but similar ratings of overprotection (10 *v.* 9.0) on the Parental Bonding Instrument. The authors suggest that close contact between parents and patients might have led to increased eating disorder symptoms or increased parental awareness and earlier referral. These findings referred to the whole eating disorder sample; no subanalyses on the ARFID group were conducted.

## Discussion

This systematic review identified seven papers that met the inclusion criteria. Four were of case reports, outlining the clinical presentation of patients aged 3–20 years from three different countries.^32,34,36,37^ Two male children^34,36^ had autism (one had a prior diagnosis and one was diagnosed during admission) and pre-existing food selectivity, but the authors did not elaborate as to if and how diagnostic criteria for ARFID applied. The authors highlighted ways by which the pandemic was considered impactful in their presentation, by either a specific fear of viral contamination by airborne virus^34^ or secondary to COVID-19-related restrictions that led to a detrimental loss of routine and structure.^34,36^ The two cases of young women are more complex, and highlight difficulties in understanding the pathology of severe weight loss in terms of differentiating between eating disorder types and organic causes.^32,37^ In these cases, because of the association between COVID-19, gastrointestinal symptoms and weight loss, the authors highlighted the complexity of diagnosis and treatment, emphasising the importance of the correct use of evaluation scales complementary to a diagnosis, and taking a multidisciplinary holistic approach with cooperation of specialists from various fields.

Three papers were found from cohort studies, all extracting data from medical records.^38–40^ Although each cohort contained patients reported as having ARFID, specific diagnostic criteria was not elaborated upon (the DSM-5 was cited in two papers). Although the numbers of cases with ARFID in all cohorts was low (ranging from 8 to 20), in the study by Otto and colleagues^38^ there was a slight increase in numbers of cases of ARFID (13 in total from the 3 years preceding COVID-19, seven in the first year of COVID-19), although the proportion of ARFID cases in the full eating disorder sample reduced slightly (from 9 to 7%).^38^ The second study found no change in ARFID referrals,^39^ from seven to seven, although two of the patients in the 2020 cohort reported that their ARFID was triggered by COVID-19. Although the final study found a slight increase in numbers of referrals with ARFID pre- to post-onset of COVID-19,^40^ this change was small (from three to five referrals). Overall, the findings from the included cohort studies are not suggestive of a difference in ARFID prevalence before and during the COVID-19 pandemic, although it is likely too early to evaluate the full impact of the pandemic.

### Eating disorders during COVID-19

As with other mental health conditions, the current review demonstrated that COVID-19 could have contributed to the development of eating disorders in vulnerable individuals, even suggesting it to be a direct trigger.^36,37^ Although Spettigue and colleagues^39^ reported that the majority of young people assessed in April to October 2020 did not view COVID-19 as triggering (60% across all cases of eating disorders, 71% of ARFID cases), for those who did, it was associated with increased risk of medical instability. Furthermore, several reasons were identified, or at least proposed by authors, across studies as to why COVID-19 might have acted as a risk factor for deteriorating eating psychopathology and adverse changes in eating and exercise behaviours, general mood and parenting. These included media representation of the pandemic, fear of viral contamination of food, changes in routine, going out to usual activities less, disruption of education and isolation, in line with previous eating disorder research.^4–6,19^

Alongside the negative effects from COVID-19 restrictions and stay-at-home orders, some studies reported on positive effects of the pandemic, with Takakura and colleagues^40^ reporting higher levels of maternal care and connectedness during the pandemic, perhaps leading to increased parental awareness and earlier referral (given the shorter illness duration upon presenting). This is similar to positive effects identified in online survey research conducted by Termorshuizen and colleagues,^19^ which could not be included because of a lack of ARFID-specific data. Qualitatively, they also identified several positive changes during the pandemic, including increased availability of social support, feeling more relaxed, increased connection with friends and family, and increased motivation to recover,^19^ yet this increased connection and time with family members has been posited as a risk for deterioration, showing individual differences.

### ARFID during COVID-19 and clinical implications

Despite the reported impact of COVID-19 on eating disorders, the current review has not suggested a significant increase in presentations of new-onset ARFID cases. A potential explanation could be that youth with body image concerns and food restriction driven by a desire to lose weight may have been more influenced by media messaging and increased media consumption during COVID-19.^4,26,41^ However, given the link of ARFID with other diagnosis such as anxiety, obsessive–compulsive disorder and autism,^26,42^ it would not be unexpected to see higher rates of distress and destabilisation among such individuals, where difficulties accessing their normal variety of foods might have been limited, changes in structures and routine leading to disruptions in normal meal scheduling (often necessary for individuals with low interest in food), and mood-related issues reducing overall appetite in already compromised individuals. These specific effects do not seem to have been reported in empirical literature this far, although all of the identified cohort studies only explored COVID-19 on broader eating disorder populations, as opposed to specifically ARFID, with little information regarding demographic or clinical details provided, making it difficult to understand the role the pandemic may have played in the illness trajectory.

Additionally, it is possible that the stay-at-home orders might have dissuaded families from attending services, leading to reduced presentation. Individuals presenting to services with food restriction and weight loss might have been misperceived as atypical anorexia nervosa, with the assumption of undisclosed fear of fatness, compromising therapeutic alliance and leading to poor treatment outcomes.^20^

Regarding the presentation of ARFID during the pandemic, the current report identified a small number of case reports linking the pandemic to presentation of cases with ARFID-like symptoms.^32,34,36,37^ However, awareness of diagnostic criteria and management of ARFID in clinical practice has been recognised to be low,^43,44^ highlighting the need for increased training. Therefore, developing and evaluating ARFID training could be a worthy area for future research.

Increased training would enable a continued focus on this poorly understood disorder. As shown by the case reports in this review, this is especially salient for those working with individuals with autism, where diagnostic overshadowing may apply.^34,36^ Likewise, it may be beneficial for those presenting with ARFID to be considered for an autism assessment if initial observations and developmental history taking are suggestive, as reported in the report from Sakamoto and colleagues.^36^ Additionally, increased training of ARFID across professions would enable greater and multidisciplinary working to help differentiate between ARFID and other medical difficulties that can present as ARFID-like symptoms, such as the gastrointestinal effects of COVID-19 as reported in the report by Borriello and colleagues.^32^

Given the serious physical implications that can ensue when the body is deprived of essential nutrients, a low index of suspicion for the disorder is warranted to allow for effective and timely treatment. The nature of ARFID is such that patients require a multidisciplinary team of practitioners to provide nutritional rehabilitation, psychological support and medical treatment.^45^ Little is known regarding how COVID-19 has affected treatment pathways for ARFID. Therefore, further studies specifically examining confirmed ARFID cases, providing an in-depth summary of typical/atypical clinical characteristics and treatment outcomes during COVID-19, would be beneficial to help us to understand the impact the pandemic has had on the development, identification, treatment and outcomes of individuals with ARFID.

Current literature on the epidemiology of ARFID is limited.^28^ Given that prevalence estimates varied greatly before the pandemic, ongoing surveillance, research and training is crucial. Furthermore, because of the complex nature of eating disorders and substantial time before seeking help and onward waiting lists,^46,47^ it is likely that it is too early to evaluate the full impact that COVID-19 has had on the presentation of ARFID.

### Limitations

Although this current systematic review is the first to examine the presentations of ARFID and ARFID-like conditions during the COVID-19 pandemic, there are several important limitations to consider when interpreting the findings. First, given the study heterogeneity, quantitative pooling (i.e. a meta-analysis) was not appropriate. The samples reported on were small (range: 1–20, total: 46), resulting in limited power to detect or report on statistically significant differences. Furthermore, the samples lacked demographic information (e.g. socioeconomic status) and representation from specific regions that were strongly affected by COVID-19 (e.g. no cohort studies from Asia), making it difficult to generalise these findings to wider populations. The lack of elaboration on diagnostic criteria mentioned for ARFID and the lack of subgroup analyses in the cohort studies, which all included individuals with other eating disorders, also made it difficult to ascertain the specific effects of COVID-19 on ARFID. Moreover, the cohort studies were retrospective in design, and it is possible that other confounding factors may have led to differences between pre-pandemic and during pandemic cohorts, such as reduced access to healthcare services.^48^ Additionally, only specialist services were studied, typically only accepting referral for severe eating disorders, which may not represent the effects of all individuals with eating disorders.

In conclusion, this systematic review explored whether the well-recognised increases observed worldwide with regard to eating disorder psychopathology and clinical presentation during COVID-19 also applied to ARFID. However, to date, this does not seem to be the case, at least with reference to published data. It is difficult to ascertain whether this is because COVID-19 has not had an effect on ARFID, or if cases remain unrecognised and/or are being perceived as or grouped with other types of eating disorders. The current review supports findings that predate COVID-19 regarding the need for ongoing surveillance, training and research into this relatively new diagnostic concept. It is likely too early to evaluate any specific impact that COVID-19 may have had on the development, identification, treatment and outcomes of individuals with ARFID, emphasising the need for continued research on ARFID and COVID-19.

## Supporting information

Maunder et al. supplementary material 1 Maunder et al. supplementary material

Maunder et al. supplementary material 2 Maunder et al. supplementary material

## Data Availability

Data availability is not applicable to this article as no new data were created or analysed in this study.
