# Supplementary material for: A systematic review of COVID-19 and the presentation of avoidant/restrictive food intake disorder and avoidant/restrictive food intake disorder-like symptoms
Source: BJPsych Open. 2024 Mar 4;10(2):e56. doi: 10.1192/bjo.2023.655 (PMC10951850; doi:10.1192/bjo.2023.655)
Supplement: Maunder et al. supplementary material 1 — Maunder et al. supplementary material [file S2056472423006555sup001.docx]

**Cochrane Library**

1. avoidant/restrictive food intake disorder OR avoidant eating OR restrictive eating OR food refusal OR calorie restrict:ab,ti,kw
2. COVID-19 OR COVID* OR corona*:ab,ti,kw
3. #1 and #2

**CINAHL**

1. (“avoidant/restrictive food intake disorder”) OR (“avoidant eating”) OR (“restrictive eating”) OR (“food refusal”) OR (“calorie restrict”)
2. (COVID-19 OR COVID* OR corona*)
3. 1 and 2

**PsychINFO**

1. “avoidant/restrictive food intake disorder” OR “avoidant eating” OR “restrictive eating” OR “food refusal” OR “calorie restrict”
2. “COVID-19” OR “COVID*” OR “corona*”
3. 1 AND 2

**EMBASE**

1. ‘avoidant/restrictive food intake disorder’ OR ‘avoidant eating’ OR ‘restrictive eating’ OR ‘food refusal’ OR ‘calorie restrict’
2. ‘COVID-19’ OR ‘COVID*’ OR ‘corona*’
3. 1 AND 2

**MEDLINE**

1. avoidant/restrictive food intake disorder [MeSH Terms] OR “avoidant/restrictive food intake disorder [Text Word] OR avoidant eating [MeSH Terms] OR “avoidant eating” [Text Word] OR restrictive eating [MeSH Terms] OR “restrictive eating” [Text Word] OR food refusal [MeSH Terms] OR “food refusal” [Text Word] OR “calorie restrict” (MeSH Terms] OR “calorie restrict” [Text Word]
2. COVID-19[MeSH Terms] OR COVID-19[Text Word] OR COVID*[MeSH Terms] OR COVID*[Text Word] OR corona*[MeSH Terms] OR corona*[Text Word]
3. 1 AND 2
