# Supplementary material for: A systematic review of COVID-19 and the presentation of avoidant/restrictive food intake disorder and avoidant/restrictive food intake disorder-like symptoms
Source: BJPsych Open. 2024 Mar 4;10(2):e56. doi: 10.1192/bjo.2023.655 (PMC10951850; doi:10.1192/bjo.2023.655)
Supplement: Maunder et al. supplementary material 2 — Maunder et al. supplementary material [file S2056472423006555sup002.docx]

**List of included papers**

Borriello G, Lavatelli L, Ruzzi F, Panariello A, Percudani ME. Case Report: COVID-19 Infection With Gastrointestinal Symptoms and Mood Disorder: Criticalities in Differential Diagnosis, Therapy and Management of Complications. Front Psychiatry. 2021 Dec 10;12.

Cao LL, Gaffney LK, Marcus C. Hypokalemia-Induced Rhabdomyolysis in a Child with Autism Affected by the COVID-19 Pandemic. Journal of Developmental & Behavioral Pediatrics. 2022 Jun;43(5):e356–60.

Otto AK, Jary JM, Sturza J, Miller CA, Prohaska N, Bravender T, et al. Medical Admissions Among Adolescents With Eating Disorders During the COVID-19 Pandemic. Pediatrics. 2021 Oct 1;148(4).

Sakamoto S, Miyawaki D, Goto A, Harima Y, Tokuhara D, Inoue K. COVID-19 phobia in a boy with undiagnosed autism spectrum disorder. Medicine. 2021 Jun 4;100(22):e26233.

Spettigue W, Obeid N, Erbach M, Feder S, Finner N, Harrison ME, et al. The impact of COVID-19 on adolescents with eating disorders: a cohort study. J Eat Disord. 2021 Dec 4;9(1):65.

Takakura S, Toda K, Yamashita M, Kitajima T, Suematsu T, Yokoyama H, et al. Potential impact of the COVID-19 pandemic on japanese patients with eating disorders -a cross-sectional study. Biopsychosoc Med. 2022 Dec 6;16(1):2.

Yazdani S, Bloomberg Z, Klauber R, and Meresh E. Avoidant restrictive food intake disorder emerging during COVID-19 pandemic resulting in superior mesenteric artery syndrome. Eating and Weight Disorders. 2022; 27:2943-2945.
